# Cultural significance of termites in sub-Saharan Africa

**DOI:** 10.1186/s13002-017-0137-z

**Published:** 2017-01-26

**Authors:** Arnold van Huis

**Affiliations:** Laboratory of Entomology, Wageningen University & Research, Wageningen, The Netherlands

**Keywords:** Ethno-entomology, Ethno-medicine, Entomophagy, Termite mounds, Religion, Superstition, Folklore, Witchcraft

## Abstract

**Background:**

The number of termite species in the world is more than 2500, and Africa with more than 1000 species has the richest intercontinental diversity. The family Termitidae contains builders of great mounds up to 5 m high. Colonies are composed of casts: a queen, a king, soldiers and workers. Some species of termite cultivate specialised fungi to digest cellulose. Termites constitute 10% of all animal biomass in the tropics. The purpose of the study was to make an overview of how termites are utilized, perceived and experienced in daily life across sub-Saharan Africa.

**Method:**

Ethno-entomological information on termites (Isoptera) in sub-Saharan Africa was collected by: (1) interviews with more than 300 people from about 120 ethnic groups from 27 countries in the region; (2) library studies in Africa, London, Paris and Leiden.

**Results:**

Vernacular names relate to mounds, insects as food, the swarming, and the behaviour of termites. Swarming reproductive, soldiers and queens are collected as food. There are many different ways to harvest them. Termites can also be used as feed for poultry or as bait to catch birds and fish. The mushrooms that grow each year from the fungus gardens on the termite mounds are eaten. The soldiers, the fungus gardens and the soil of termite mounds are used for multiple medicinal purposes. Mounds and soil of termites have numerous functions: for geochemical prospecting, making bricks, plastering houses, making pots, and for storage. Termite soil is often used as fertilizer. The act of eating soil (geophagy) among women, especially those that are pregnant, is practised all over Africa. The mounds can serve as burying places and are often associated with the spiritual world, especially containing the spirits of ancestors. Termites also play a role as oracle, in superstitious beliefs, in art and literature.

**Conclusion:**

The following characteristics make termites so appealing: the dominance in the landscape, the social organization, the destructive power, and the provision of food. The study shows that termites play a major role in peoples’ lives, in physical as well as spiritual aspects.

## Background

The number of termite species in the world is more than 2500 (p. 32, [1]); Africa with more than 1000 species has the richest intercontinental diversity [2]. The family Termitidae contains builders of great mounds, e.g. *Macrotermes* spp. up to 5 m high and 12 m across (p. 67, [1]) and more than 1.800 species have been described, many from Africa. A queen belonging to the family of Termitidae can grow up to 6 cm of length and produce 10 million eggs per year. The colonies are composed of casts: a queen, a king, soldiers and workers. The future queens and kings have wings and they depart from the colony at the start of the rains after the dry season. They mate and shed their wings to start new colonies. Termites play a major role in recycling wood and plant material. They tunnel the soil, making it porous, aerated and enrich it with minerals and nutrients. Some species of termite practice fungiculture. They maintain a “garden” of specialised fungi of the genus *Termitomyces* which are eaten [3].

In the tropics, as a whole, termites are thought to constitute 10% of all animal biomass (up to 95% of soil insect biomass), and to impact carbon mineralisation (decomposition) to roughly the same extent as all mammalian herbivores and natural fires [4].

The purpose of the study was to make an overview of how termites and termite mounds are utilized, perceived and experienced in daily life across sub-Saharan Africa. This was based on interviews in 27 African countries and literature reviews.

## Methods

The information was collected by reviewing the literature and by personal interviews. The interviews were conducted in the years 1995 and 2000 in Africa and concentrated on the traditional, nutritional and medical uses of arthropods and their products as well as on their role in religion, witchcraft, art, song, music, dance, children’s games, mythology and literature. A part of the results obtained in 1995 on insects in general has been published [5], while the part on edible insects in general over both years (1995 and 2000) has been published in 2003 [6].

The total number of people interviewed was 302 from 27 different countries in sub-Saharan Africa of whom 21 were resource persons (experts without recorded ethnic affiliation). From 5 other respondents, the ethnic group was unknown. The total number of ethnic groups was 121, excluding Zanzibar and Madagascar where the ethnicities were not recorded.

The following part presents a listing of countries involved and, for each of those, the number of persons interviewed with, between parentheses, a breakdown according to ethic group. RES stands for resource person.

Benin-14 (Bariba-1, Fon-4, Goun-1, Nagot-6, Popo-1, Tori-1), Burundi-2 (Hutu-2), Burkina Faso-5 (Mossi-4, Fula-1), Cameroon-30 (Bamileke-14, Bafia-1, Bakoko-1, Bakossie-1, Banen-1, Bani-Pahuin-1, Bassas-2, Beti -Eton-1, Beti-Ewondo-1, Bolous-1, Matha-1, Tikar-1, Wimboum-1, Yambassa-1, RES-2), Central African Republic (CAR)-2 (Gbaya-1, Kari-1), Chad-17 (Arabe-1, Goulaye-2, Kanembou-1, Mbaye-2, Ngambaye-7, Sara-Kaba-1, Sara-Niellim-1, Tupuri-1. Wadai-1), Democratic Republic of Congo (DRC)-2 (Mbochi-1, Teke-1), Gambia-2 (Jola-1, Mandinka-1), Guinee-Bissau-1 (Balanta-1), Kenya-13 (Kalenjin-1, Kamba-4, Kikuyu-2, Luo-4, Meru-1, Somalian-1), Madagascar-24 (24), Malawi-1 (Chewa-1), Mali-10 (Fula-1, Mande-Malinke-1, Mande-Mandinka-1, Sarakolé-1, Senufo-2, Songhay-3, Tuareg-1), Mozambique-8 (Bitonga-1, Makua-1, Nchope-1, Shona-1, Tsonga -Rhonga-2, Tsonga-Shangana-1, Tsonga-Tswa-1), Namibia-1 (Damara-1), Niger-15 (Djerma-1, Hausa-9, Kanuri-1, Songhai-4), Nigeria-18 (Ebibio-1, Ebira-1, Yoruba-15, Unknown-1), Rwanda-1 (Kiga-Toro-1), Senegal-17 (Bainuk-1, Diola-4, Fula-1, Halpulaar-2, Lebu-1, Serer-3, Wolof-5), South Africa-6 (RES-6), Sudan-23 (Dongolawi-1, Fula-1, Gaälien-3, Kambari-Abadi-1, Kawahla-1, Kuku-1, Mahas-1, Nubian-1, Nubian-Mahas-1, Rubatab-2, Tunyur-1, RES-5, unknown-4), Tanzania (Chaga-7, Digo-1, Iraqw-3, Iramba-1, Mwarusha-2, Pare-1, Rangi-1, Sukuma-2, Zanaki-1), Togo-11 (Akebu-1, Ewe-5, Cotocoli-1, Kabye-1, Mina-1, RES-3), Uganda-15 (Acholi-1, Banyankole-1, Bunyoro-1, Busoga-1, Ganda-7, Langi-1, Luo-2, Nyoro-1), Zambia-22 (Bemba-1, Ila-1, Lovale-1, Lozi-2, Lunda-1, Namwanga-2, Nyanja-Chewa-1, Tonga-10, Tumbuka-1, RES-2), Zanzibar (9), Zimbabwe-13 (Ndebele-1, Shona-9, Zezuru-1, RES-2). Names of ethnic groups were checked, mostly in Wikipedia [7] and the Joshua project [8].

Most of the people interviewed were scientists or technicians trained in entomology. This was done by visiting entomological groups of universities and (inter)national agricultural research institutes, plant protection services, museums or crop protection projects. It was tried to interview most of the staff of these organizations (often arranged by the responsible officers). The age of the persons interviewed varied between 25 and 65. Most of the informants were male reflecting the gender composition of the organizations. A few times people were interviewed in villages who had no entomological background. This proved to be a challenge because of language and confusion about the insect species. Twenty-one of the respondents acted as resource persons on special subjects (for example experts on termites or insects as food or medicine) or with special positions (professors, heads of organizations, shamans, museum directors, and priests). In these cases, the ethnic origin of the person who provided the information was not considered relevant.

The author used a list of issues to be covered in the interviews. Some informants got the list before the country was visited. A few times the informants questioned elders, family members and acquaintances before my arrival and provided me with this information. The national libraries and university libraries in London and Paris, the library of the African Studies Centre in Leiden, the Netherlands, and some libraries of the countries visited were consulted. The literature consulted was mainly of anthropological nature.

Findings for a particular country or a certain tribe were only reported if information was received from more than one informant, or if the information given during interviews was confirmed in the literature. Particulars on the respondents’ countries and tribes are mentioned to specify the sources of information. They cannot be used for establishing correlations between ethnicity and information provided. The qualitative character of the information provided is emphasized.

## Results and discussion

### Classification

Local names for termites differ from taxonomic classification. For example, the Mossi population of a village in Burkina Faso used vernacular species names to categorize different termite species: five for *Macrotermes subhyalinus*, four for *Trinervitermes* sp. and five for four other termite species [9]. The vernacular names referred to the shape of the mound, time of swarming (nuptial flight) or to termites’ behaviour, colour or dangerousness. In Burundi (Hutu) a difference was made between the termites that can be eaten, ‘Iswa’, and the termites ‘Umuswa’ that destroy wood and furniture. In Zambia, the Nkangala and Nkoya define ethnospecies of termites primarily by combining morphological, behavioural, consanguinal and utilitarian traits (pp. 169-170, [10]). The latter one is the most important as all are used either for food, medicine or another practical purpose or they have a bad reputation (inflicting pain or material damage). In the latter study, no exact overlap was found between the termite ethnospecies and the scientific species. That is to say casts, sexual stages and juvenile forms of a species are not known, and therefore not classified into one ethnospecies, while relations between ethnospecies are structured according to kinships and class terms used in human relations.

### Entomophagy

#### General

Entomophagy may have been a part of early hominid diets. Backwell & d’Errico [11], analysing bone tools from the Lower Palaeolithic sites of Swartkrans and Sterkfontein in South Africa, concluded that they were used by *Australopithecus robustus* to dig into termite mounds, and not, as was supposed earlier, to dig for tubers. Also Lesnik [12] concluded that Plio-Pleistocene hominids used a combination of soldiers and alates of the genus *Macrotermes* due to their significant amounts of energy-yielding nutrients and potential role as a critical resource for supporting larger-brained hominids. In the 1980s, studies on wild chimpanzees from western, central and eastern Africa showed that the fishing technique to extract termites from *Macrotermes* spp. mounds was the most popular [13, 14], and Joulian & Roulon-Doko [15] drew our attention to the similarity of termite extraction by humans.

#### Reproductives

Winged termites are popular food in Africa. This concerns the reproductive caste, which have a nuptial flight after the first rains following the dry season. After the flight, the reproductives (alates) shed their wings. There are many ways in which these insects are collected, depending on the termite species and the time that they emerge (pp 116-125, [10]), [16]. During expected emergence of the alates, mounds are checked for appearance of emergence holes. I was told that in Zimbabwe (Shona) and Zambia the most important *Macrotermes* spp. eaten are: *M. falciger*, *M. michaelseni*, *M. natalensis*, *M. sybhyalinus*, and *M. vitrialatus*. For those that emerge during the night, the most common way to collect them is to place a lamp above a bowl of water. They are attracted by the light and fall into the water from which they are scooped. A hole can be dug near the termite mound and a fire (e.g. of grass bundles) is lit nearby. This can also be a trench around the termite mound (Sudan). The attracted termites are then swept into the hole. Sometimes the chamber inside the termite mound (alates group themselves in special chambers near the periphery of the nest for several days or weeks) with all the reproductives is dug up (Cameroon: Bamileke; Sudan: Dongalawi). A tent can be put up over the termite mound made from leaves of Andropogon grass (Chad: Tupuri), manioc (DRC: Mbochi), *Eriosema shirense* (Leguminosae-Papilionoideae) [17], banana, bamboo, raffia or straw (Burundi: Hutu; Cameroon [18], Bamileke; Kenya: Luo; Tanzania: Chaga, Marusha; Uganda: Acholi, Ganda) or plastic (Zimbabwe Shona). Some exit holes may be plugged in order to assure that they exit just from a few. The soil is often covered with banana leaves (Kenya: Luo; Uganda: Ganda, Nyoro) to facilitate sweeping. From holes made in the tent illuminated by an artificial light source outside, the termites are caught. Another way to make them emerge is by pounding rhythmically on the soil with stones and sticks to simulate heavy rainfall and sometimes simultaneously water is poured over the mound to raise humidity (Cameroon: Bamileke; Kenya: Luo, Somali, Tugen mentioned by Ogutu [19]; CAR: Gbaya; Uganda: Acholi, Ganda, Langi, Busoga (also [20]); Zambia: Nyanya). In the Republic of South Sudan this may be accompanied by songs such as ‘*anyeku me kotu*’ which means ‘*come out in numbers like raindrops*’ (p. 159, [21]). Another way to trigger emergence is to use an overturned calabash and then tapping on it (Uganda: Kuku; CAR: Gbaya [22]) or pound with a stone on a bigger one which is on the ground (Kenya: Luo).

Winnowing is done after frying to get rid of the wings. Termites are fried without oil as they have lots of fat. The oil that remains is often used as cooking oil; see also Tihon [23]. After frying the termites can be conserved for 3 to 7 days. They can be dried and then conserved for several months (Cameroon: Bamileke; CAR: Gbaya; Chad: Ngambaye). In Uganda (Ganda, Langi) for long conservation they should be kept in banana leaves above the cooking fire or to be re-dried periodically (in the sun). Another way is boiling in salt water (Kenya: Luo), or vapour cooking in a banana leaf (Cameroon: Bamileke; Uganda: Bunyoro, Ganda), after which the termites are dried and stored. Termites can be crushed to make flour (Sudan) and made into a cake (Cameroon: Banen; Congo: Teke; CAR: Gbaya, Kari) or used in a tomato stew (Uganda: Ganda). In Uganda a sauce, ‘*Ekipooli*’, is made from termites which have first been steamed and dried [24]. Termites are considered to be very nutritive and they were compared with the first milk given by a cow after delivery (many antibodies) and drinking it for several days would make the winkles in your face disappear (Chad: Wadai). The termites can be eaten with some salt, pepper, tomatoes, onions or with a sauce of taro (*Colocasia esculenta*) (Cameroon: Bamileke) or macabo (*Xanthosoma sagittifolium*) (Cameroon: Banen).

In Zande, an area in the south of the Sudan, north of Zaire and south-east of the Central African Republic, the gathering of wild produce such as honey, yams or wild fruits, is done by men and mushrooms and caterpillars are collected by women [25]. However, catching termites is done by the whole family. There are several termite species in which the reproductives appear at different times (April/May and mid-August to mid-November). The harvesting of termites may take about a quarter of people’s time. All, except very small children and some older relatives who remain to look after them, leave home in the evening armed with baskets of two kinds – one for catching and one for storing the caught termites, and grass torches (stems of the thatching grass *Hyparrhenia* spp., which survived early bush fires as they were too juicy to burn).

Termite mounds are often not owned and therefore termites and the mushrooms can be collected by everybody (Togo: Kabye; Tanzania: Rangi).

#### Soldiers

Soldiers of termites are eaten (Benin: Fon; Burkina Faso: Burundi: Hutu; CAR: Gbaya (see also Roulon-Doko [18], Gharé; Chad: Ngambaye; CAR: Gbaya; Burundi: Hutu; Kenya: Luo; Nigeria: Yoruba; Sudan: Dongolawi; Uganda: Langi, Luo; Zimbabwe: Shona). However, a number of informants told specifically that they are not eaten by their ethnic group, which is confirmed by Silow (p. 91, [10]). The collection is often done by children [16], such as the termite clubs in Uganda (pp. 144-149, [26]). The most common way is to break a part of the termite mound, insert a grass stem, such as *Panicum maximum* or *Cylindrica impericum* or reeds from the river, into the hole of the mound. The soldiers will bite in the stem after which they will be stripped into a container with water. Sometimes only the heads of the soldiers are eaten (Uganda: Langi, Luo; Sudan: Kuku), which are pounded into a cake (Kenya: Luo; Uganda: Langi). They can also be fried whole with salt and water and cooked with a paste of groundnut (peanut butter). The abdomen is said to be bitter.

#### Queen

It is difficult to get to the queen and often a whole termite mound has to be demolished in order to reach the queen chamber. Many interviewees indicated that the queen is not eaten. However, when it is eaten it is often for special reasons, e.g. for malnourished children (Zambia), Cameroon [27] or when ill (Chad: NGambaye). It is also considered that when somebody eats the queen he/she will be more respected (Mali: Milinke). For example, the royalties of the Yoruba in Benin believe that it will assist them in becoming more respected by the people [28]. Among the Haya in Tanzania and the Ganda in Uganda only the king or chief is allowed to eat the queen (pp. 93-94, [10]). The queen is presented to the head of the village as a sign of respect (Nigeria: Ebibio; Uganda: Ganda). The queen is taken from the queen chamber and roasted in a leaf over charcoal or the whole queen chamber is put in the fire and roasted for 10-30 min.

For women who have difficulties in getting a child, eating the queen gives a higher chance of becoming pregnant (Nigeria: Yoruba; Togo: Kabye). The queen maybe eaten in order to have a higher chance of getting twins, not only for humans but also for goats (Sudan: Kuku). In several countries the queen is eaten as an aphrodisiac (Benin: Nagot; Senegal: Wolof; Togo: Kabye; Zimbabwe: Shona). The content of the queen is put on the skin as a cosmetic in order to let it shine (Kenya: Kikuyu). The queen is used to attract customers in the market (Nigeria: Yoruba). Similarly, hunters wash themselves with an extract from the queen in order to attract wildlife (Senegal: Diola).

### Termites as feed for animals

#### Chickens

Chickens or guinea fowls do not have access to termites, which are protected by the termite mound. Therefore, farmers may break small termite nests (*Microtermes* spp.) or parts of termite mounds to feed the chickens or chicks. This is very common practice and mentioned by at least half of the informants in West Africa, but also from Burundi, Cameroon, Central African Republic, Congo, Madagascar and Tanzania. The termite species mainly used are those of the genera *Cubitermis* et *Pseudoacanthotermes*. One informant from Togo (Akebu) told that you have to careful with a species of *Trinervitermes* as it may kill the chicks; from Burkina Faso, the same is reported for this species [9]. There is a semi-domestication method to collect termites as feed. Dry stems of sorghum or other cereals, dry maize cobs, are put in a clay pot termed “canari” (i.e. a spherical clay container, Ø = 0,1-1 m, with a wide opening used in western and central Africa primarily to store and cool drinking water, and also for cooking) [29, 30]. Water is added and the clay pot is turned upside down with the opening on a termite gallery. The microclimate within the pot is ideal for the termites. After 3 to 4 weeks the number of termites is considerable and the pot is emptied for the chicks. Termite meal can also be used.

#### Bait

Termites caught in the wild can be used to catch fish and birds. Silow [10] reported from Zambia the use of snouted termites (*Trinervitermes* spp.) as fish bait in conical reed traps and as bait to attract insectivorous birds (such as guinea fowl, francolins, quails and thrushes). The birds were caught by setting a snare across the broken top of a termite mound, where soldiers mass for hours. Such a trap on termite mounds was mentioned by informants from Congo (Teke). Fishermen use termite larvae as bait (Chad: Ngambaye, Sara-Kaba; DRC: Teke).

### Termites as medicine

Soldiers have a medicinal use. For surgical purposes, they are used to suture a wound (Rwanda: Toro). The mandibles of the soldiers are applied to the edges of the skin which are drawn together over the wound. When the soldiers bite, their bodies are snipped off. The row of mandibles is left in place until the wound heals. Ground soldiers are also rubbed into cuts made in the forearm in order to make somebody a good boxer (Zambia: Tonga). The ground-up mound material is used as a paste to cure skin diseases (Togo), to treat swelling of the feet (Togo: Mina), to cure an abscess (Benin: Nagot; CAR: Gbaya; Chad: Sara-Niellim; Gambia: Jola; Sudan), to use as plaster (splint) when having a fracture (Chad: Mgambaye; Mali Songhay), and to cure angina (Senegal: Fula), an inflammation of the parotid glands (parotitis) (Benin: Bariba, Fon, Nagot; Cameroon: Bakoko), tonsillitis (Sudan; Tanzania: Iraqw) or swollen udders of cows (Tanzania: Mwarusha). It is used when a child has fever (Tanzania: Digo, Mwarusha). In Nigeria soup of the termite *M. nigeriense* is used for pregnant women to assure a safe delivery of the baby [31]. The termite soil can serve as a carrier for medicine (the soil preserves it) (Uganda: Bunyoro). Parts of the fungus garden are used as a cure, but together with the fruit of the plant “Akika” (*Lecaniodiscus cupanioides*) of the family Sapindaceae (leaves, roots, young shoots, seeds, stem-bark) against fever, burns, liver abscesses, jaundice, cough, malaria; or as a purgative or aphrodisiac [32]. The healing power of termite soil was mentioned in a book from Liberia (p. 328, [33]). Among the Bafia in the centre province of Cameroon, the soil of mounds from two termite species is used as medicine: 1) *Nasutitermes* sp. - the vapour from boiled soil to treat eye problems; and 2. *Bellicositermes* sp. - a water mixture of soil and cola (*Cola acuminate*) nut against bleeding during pregnancy or a paste for the baby skull at birth in case of congenital hydrocephalus (excessive cerebrospinal fluid) [34].

In Benin (Goun), a piece of the fungus comb is ground and given with honey to children to stimulate the memory. Informants from Benin (Tori) told me that from the termite mound, nine pieces of *Imperata cylindrica* leaves and nine fruits of the ginger species *Aframomum melegueta* (Zingiberaceae) should be given to male children to stimulate their memory; the number in case of female children would be seven of each plant (Benin: Tori). The fruit is known from Benin to be used against headaches [35]. The Nagot in Benin told me that somebody with epilepsy should climb the termite mound, take soil from the top, put it in a calabash with water and drink it.

### Termite mounds

#### Geochemical prospecting

Termite mounds are used to explore for gold, zinc, uranium, and other metals [36–38]. Termites can dig up to 10 m underground, even to 70 m [39], either to avoid drought, to obtain clay for building purposes or for moisture. They then ingest and bring the new deposits to the surface. They do not concentrate metals in the bodies, they actively rid their bodies of excess metals. These excretions of mineral deposits in the mound are used commercially by mining companies to determine the location of gold and other mineral deposits. The technique is an alternative to invasive and expensive drilling methods.

#### Building material

In many parts of Africa soil from deserted termite mounds is used for house construction, e.g. in Zambia (pp. 86–88, [10]). In Togo termite soil is used to make furnaces (Akebu, Cotocoli, Ewe, Kabye), while Iroko [28] indicated that old termite mounds (either the soil or the whole termite mound) in Benin have been used by blacksmiths to extract minerals such as iron. It is used as a kind of plaster for huts and for granaries to make the walls more resistant and smooth (Burkina Faso: Mossi; Chad: Goulaye, Ngambaye, Sara-Kaba; Mali: Mande-Malinke, Mande-Mandinka, Sarakole, Songhay; Mozambique: Makua, Nchope, Tsonga-Rhonga; Niger: Hausa; Tanzania: Chaga, Iramba, Iraqw; Togo: Akebu, Ewe, Kabye; Uganda: Bunyoro, Busoga, Langi, Luo; Zambia: Lozi, Nyanya; Zimbabwe: Ndebele). Likewise, the floor is done this way (Burkina Faso: Mossi), and mixed with cow dung in order to make it really smooth (Mozambique: Tsonga -Changana, Tsonga-Rhonga; Sudan: Kuku; Zambia: Lovale, Lozi, Tonga; Zimbabwe: Shona). The floor of Nelson Mandela’s elderly house was also smoothened by mixing termite soil with cow dung (his book ‘Long Walk to Freedom’ [40]). The wall of the house is often done by mixing termite soil with shea butter and cow manure to prevent cracking so that mosquitoes do not enter the house (Benin: Bariba; Tanzania: Iraqw). In Mozambique (Tsonga-Tswa), a liquid from the cashew nut shell is used as wood preservative as it acts as a termiticide [41]. In Niger (Djerma) I was told that a piece of the termite mound is used to filter water.

Termite soil is used for cooking pots (CAR: Gbaya; Burkina Faso: Mossi; Chad: Ngambaye; Mali: Sarakole; Mozambique: Makua; Sudan; Tanzania: Mwarusha, Zanaki; Uganda: Busoga; Zambia: Lozi, Tonga; Zimbabwe: Shona), for storing water (Sudan) or for cooking leaves or roots for medicinal use. Whole small termite mounds are used as receptacles, after removing the contents. In Chad (Ngambaye) it is then used to cook peanuts.

Soil from termite mounds is used to make bricks (Burundi: Hutu; Cameroon: Bamileke, Beti-Eton; Burundi: Hutu; CAR: Ghare; Chad: Goulaye, Mbaye, Ngambaye, Sara Kaba; Kenya: Luo; Mali: Senufo, Songhai; Mozambique: Makua; Nigeria: Yoruba; CAR: Kari; Senegal: Bainuk, Halpulaar, Serer; Tanzania: Mwarusha, Iramba, Zanaki; Uganda: Bunyoro, Busoga, Langi, Luo; Zambia: Lovale, Tonga; Zimbabwe: Shona).

Mangrove wood boards are superior for supporting the roof of houses to those made of the Palmyra palm, *Borassus* spp., since mangroves are not preferred by termites (Senegal: Diola). The same informant indicated that shells (from the sea) are ground and mixed with soil (‘*banco’*) for house construction. This prevents termites from entering the house. From an informant in Zambia, I heard that *M. falciger* and *Odontotermes* spp. cause widespread damage to buildings in Zambia and that the tree *Pterocarpus angolensis* (Fabaceae) is used as building material as it is resistant to termites [42, 43].

#### Fertilizer

Soil of termite mounds are normally nutrient-rich, in particular of calcium, magnesium, potassium and sodium and available phosphorus. Besides, the mound soil is characterized by high fractions of clay, silt and fine sand as well as organic matter. To use this soil as fertilizer can lead to a three-fold increase in yield. Farmers then grow the crop in this nutrient-rich soil, in which the crops grow well but where the termites are also abundant (p. 79, [5]), [44]. Sileshi et al. [45] give an overview of how farmers in Africa grow crops on or near termite mounds and how they spread the nutrient-rich soil from termitaria in their field. In flat areas of low fertility in Zambia, especially if they are liable to flooding, farmers cultivate large mounds for the crops while in Malawi *Macrotermes* mounds are selected for tobacco gardens [46]. Mounds play an important part in the natural generation of forests (the evergreen shrubs and trees colonize these mounds and escape the worst effects of grass fire) [46].

Iroko [28] mentions how in Benin the decision to settle is influenced by the presence of termite mounds: “*Under the reign of Adandozan (1997–1818) of Abomey, and old migrant called Dandji, left Ato-Agokpou in Togo and settled definitely on a site where abundance of termite mounds was for him a foreteller of prosperity”*. This was confirmed by informants (Mali: Mande Madinka, Mande-Malinke). Informants told that plants always grow better near a termite mound (Benin: Goun, Nagot; CAR: Ghare; Chad: Ngambaye; Mali: Sarikole; Tanzania: Zanaki; Togo: Ewe) and that the soil can be used as a fertilizer (Cameroon: Bamileke, Bolous; Senegal: Diola; Tanzania: Chaga, Iraqw; Zambia: Tonga; Zimbabwe: Shona). Termites are also used for soil rehabilitation, e.g. the “zai” system in Burkina Faso [47] in which termites are crucial in water retention by incorporating organic matter into the soil [48].

#### Geophagy

Geophagia is the practice of eating earth or soil-like substrates such as clay or chalk. Accumulation of minerals such as calcium, phosphorus and potassium occurs in the mounds. Elephants eat the termite soil and is considered a kind of salt lick (p. 80, [5]). An informant from Chad (Daye) mentioned this for other wild animals and cattle. Most informants indicated that pregnant women eat soil and very often from termite mounds or termite runways. Sometimes the soil from huts is used as these are constructed from termite mounds. In Sierra Leone the soil may be dried and smoked over the fire before being used [49]. The most frequent reason for eating termite soil was that women feel an urge to do so. Some Informants indicated that it was necessary for the growth of the foetus and others that it provided iron which is present in the soil. This is confirmed by a study reviewing geophagy by pregnant women [50]. Soil may provide 14% of the recommended dietary allowance of iron in pregnancy. This study among pregnant women in a number of African countries reveals a prevalence of geophagy between 15 and 84% percent. In western Kenya, it was about 50% and half of those preferred termite soil [51].

#### Hunting

Abandoned termite mounds may contain small mammals, such as pangolin (DRC: Teke) and are therefore favoured by hunters (Chad: Ngambaye. Mbaye; Zambia: Tonga). However, those mounds can be dangerous as they may shelter snakes (Chad: Ngambaye; Cameroon: Bamileke; DRC: Mbochi; Kenya: Luo; Madagascar; Tanzania: Chaga; Madagascar; Niger: Hausa; Uganda: Kuku). Termite mounds are damaged to attract birds which are then caught in a trap (DRC: Teke), e.g. *Quelea quelea* (Tanzania: Sukuma). From a recent disturbed part of a termite mound, the fresh clay is taken from the repaired part to make balls in order to shoot birds with a catapult (Uganda: Luo). The termite mound is used as a lookout to see where wildlife, cattle or other persons are (Tanzania: Iraqw, Mwarusha; Zambia: Tonga). Termites are used as a bait for fish (Pemba; Zambia: Tonga), and Silow (p. 149, [10]) indicates that in Zambia snouted harvester termites (*Trinervitermes* spp.) are used as such.

##### Storage

In Cameroon (Bafia, Banen, Tikar) kola nuts (fruits of the kola tree) can be stored in termite mounds: termites would only attack the outer part of the cola nut. It can be stored as such for years and it would enhance the quality of the nut. In Cameroon cola weevils *Balanogastric kolae* and *Sophrorhinus* spp. (Col.: Curculionidae) are apparently major pests of cola nuts, prior to harvest and during storage and Facheux et al. [52] *indicated* also that one of the traditional practices for protection against these pests is the burial of the nuts in termite mounds.

##### Burying places

From Zambia (Nyanja, Tonga) and Zimbabwe (Shona) (see also Gelfand [53]), informants told a number of times that abandoned termite mounds are favoured burying places. As the plains often get waterlogged (muddy), people prefer to bury the dead on hills or mounds. The Baoulé in Côte d’Ivoire used to bury people who died of leprosy in large termite mounds (pp. 84–85, [54]). The Agni people of Côte d’Ivoire do the same, among other to prevent people from walking over the grave with a risk to get the contagious disease (pp. 168–169, [55]). A similar use was mentioned from Benin by Iroko [28]. Weidner [56] reports from southern Africa that the Khoikhoi bury the dead in large termite mounds. Because of hunters and their relation with termite mounds, the Ndembu (Lunda) hunters from the Central African Republic may be buried in termite mounds (p. 295, [57]). However, there is some ancestral wisdom involved: termites humidify the earth in order to build large termite mounds, but they have no water, so God himself provides it. Termite mounds were also associated with the dead as in graveyards often many mounds are present (Mali: Senufo).

#### Edible mushroom from termite mounds

Edible mushrooms of the genus *Termitomyces* arise from the fungus gardens of the termites [7]. There are about a dozen species and most are highly valued as food [58] or as medicine [45]. They are harvested and marketed for consumption. For the ethnic groups the Baoulé and Abbey In central and southern Côte d’Ivoire, these mushrooms are a key source of cash income, especially for women (traders) and the farmers (harvesters). However, overexploitation is threatening their persistence as well as that of the dependant termite species, and developing sustainable harvesting practices will be necessary [59]. Some informants told about the eating of mushrooms, especially the very big ones, on termite mounds (Cameroon: Bamileke, Bani-Pahuin; Mozambique: Makua). From Nigeria, *T. robustus* is very popular as food, and the cap can become almost 20 cm in diameter [58, 60]. Contrary to the termites, the mushrooms belong to the landowner (Cameroon: Bassas). However, according to an informant from Congo (Teke) the mushrooms can be harvested by anybody.

#### Bioluminescence associated with termites

One informant from Cameroon (Bamileke) told about light coming from wood infested by termites. I could not get a confirmation that this happens in Africa. It is known from central Brazil that the beetle *Pyrearinus termitilluminans* (Col.: Elateridae) lays its eggs in the sides of termite mounds [61]. When the young hatch, they glow with a green light. The larvae are carnivorous and their luminescence attracts termites and other insects. In one publication, another species of the same genus *P. fragilis* was found in termite-infested wood, which appeared like little points of blue light [62]. However, another explanation would be that it concerns luminescent fungal mycelium growing on the light- emitting wood [63].

### Religion and superstition

#### Religion

Termites mounds seem to be associated either with ancestors, devils, spirits, witches or ghosts (French: génie) (CAR: Gbaya; Chad: Arabe, Ngambaye; Mali: Songhai; Niger: Djerma, Hausa, Songhai; Senegal: Wolof; Uganda: Luo; Zambia: Tonga; and Benin [28] and Gabon (p. 96, [10]). Even children are told to greet their grandparents when passing a termite mound (Mali: Songhai). In all cases often offers are given (Uganda: Ganda), e.g. rice or millet (Mali: Songhai; Senegal: Halpulaar, Serer, Wolof), cheese (Benin: Nagot), palm oil, alcohol (Togo: Akebu) or cola nuts (Niger: Songhai). Certain ceremonies can be performed in particular when one wants to put a spell on somebody who has done wrong (Tanzania: Iraqw). This is necessary if somebody is ill (Mali: Songhai) or when possessed by a bad spirit (Mali: Senufo). In Mali (Songhai) when somebody is possessed by the devil (Bori) a traditional string instrument played like a violin (Goge) is used near the termite mound and offerings (like kola nuts or sugar) need to be make. Madness is regarded by the Ndembu (Lunda) from the Central African Republic as an affliction by an ancestral spirit and needs to be treated on a termite mound (p. 322, [57]). Small termite mounds with evil spirits in them are brought to a certain site for driving the spirits out of someone who is suffering from them (Uganda: Langi). It was mentioned that the washing water of an ill person is put by a healer on the termite mound (Mali: Mande-Malinke). The same person mentioned that between Bamako and Sikasso, the circumcised wash themselves on top of the termite mound after the operation. Turner (p. 217, [57]) comments from the Ndembu (Lunda) in the Central African Republic that circumcised boys had to put their penis in the smoke from the bark of the tree *Brachystegia woodiana* on top of a termite mound. Washing on a termite mound is also done in order to be better protected (Senegal: Halpulaar). Because termite mounds are inhabited by either ancestors of spirits, termite mounds should not be destroyed (CAR: Gbaya), but rather respected (Senegal: Serere) or avoided (Niger: Djerma; Senegal: Wolof), particularly during the night (Mali: Songhai; Niger: Hausa). Children should not be playing near termite mounds (Chad: Arabe). Walking on a termite mound is not even recommended as one may become ill (Niger: Hausa). One even mentioned that it is dangerous to look into the chimneys of termite mounds as one may become epileptic (Senegal: Bainuk). Several informants from Madagascar mentioned that you should not urinate on a termite mound as something may happen to your testicles or penis. Medicine men and witchdoctors are often involved (Mali: Songhai). Things left on the termite mound should not be taken as bad spirits will follow someone (Niger: Hausa). People who can transform themselves into animals may hide in a termite mound (Cameroon: Bamileke).

In the narratives of the San of southern Africa, termites were the first meat that God gave to humans, before all other animal meat was created. The flying termites were associated with supernatural creative powers, and creation stories of the San relate that the first humans came from a termite nest, considered to be God’s house. That is probably the reason that in rock art of the San (Bushman) north of the River Limpopo in Zimbabwe termites’ nests are depicted [64].

Among the Azande each family has permanent ownership of the termite mounds, and even during resettlement when people move some distance away, these rights are not transferred [25]. This ownership gives the impression of the mound’s sacredness. This is demonstrated by the fact that a termitarium is sometimes used as a place to put a pot containing the ancestor’s spirits. This is done during rituals for blessing either a crop or ceremonial food and drinks. In a sacred forest the termite mounds are not touched (Benin: Fon, Popo).

The Azande from the Sudan use termites as oracles [65]. They use two branches from different trees (*dakpa* and *kpoyo*) and insert them into a termite mound. A question is asked in the evening and the next day the answer is deducted from the extent to which the termites ate from either *dakpa* or *kpoyo* or from both.

There are quite some stories about the rainbow, a snake and termite mounds. For example, the rainbow comes from a termite mound and prevents that it rains (Chad: Ngambaye; Mali: Sarakol; Niger Hausa; Uganda: Kuku; Zambia: Tonga), also believed by the Kuranko from Sierra Leone and Guinea [44]. The Ewe from West Africa believe that the rainbow was the great serpent Anyiewo (also spelled Ayido Weddo), who devours any person that he touches; the serpent lives between rainstorms in termite mounds, so they have a dread reputation [66]. The Nkoya in Zambia classify the rainbow as a snake (Nkongolo) which migrates between termite mounds (p. 97, [10]).

To celebrate twins, offerings are made on the termite mound, e.g. yam and red palm oil or guinea fowl eggs (Benin: Bariba). The umbilical cord of twins is put in a vase with two openings and placed in a kind of shelter from twigs and leaves of a special tree on a small termite mound; dancing takes place (Uganda: Langi, Luo). The same procedure is followed for babies born very prematurely. The small termite mounds are considered to harbour spirits.

From Sudan (Dongolawi, Mahas) informants told about the fate of the prophet Solomon, who died while resting on his cane watching the work of Jinns (supernatural creatures). When the stick was eaten, he fell over, and only then it was realized he was dead. The termites would not have eaten the stick when realized that it was the cane of the prophet. This is from the Quran Chapter 34 Surah Saba verse 14: …. *nothing showed them (the Jinns) his death except little creatures of the earth which kept gnawing away at his staff*…

#### Superstition

It is difficult to make a separation between religion and superstition. In general, the first is based on faith, while superstition is based on myth, magic, or irrational thoughts. For example, when a termite mound appears in the house, one should consult a witch doctor (Guinee Bissau: Balanta). From Uganda (Ganda) it was mentioned that termites are used to avoid misunderstanding and disagreement among wives. For example, if one has two wives, two termites are crushed into powder which is mixed with soil from an abandoned termite mound. The dried mixture is then dissolved into water on a broken clay pot piece and given to both wives while saying: ‘there should be understanding between these two, as there is good understanding between 2 termites’.

Informants from Tanzania (Chaga) told that the Sukuma tribe, especially those from Sinanga, Mwanze and Tabora, predicts the season by the frequency and the duration that the termites come out flying.

Several times informants told that eating a certain small termite species makes you deaf (Kenya: Luo; Uganda: Luo; Zambia: Tonga; Zimbabwe: Shona, Ndebele). In Zambia, this is reported to be believed among the Luchazi, Lovale and Chockwe and it seems to concern Termitinae and Nasutiterminae; the translation of the vernacular name is ‘ear clogger’ (pp. 151–152, [10]). Roulon-Doko [22] reports from the Central African Republic that the Gbaya do not eat a small termite species for this reason; the species is used to feed chickens.

In Zambia (Tonga) ground jaws of soldiers are rubbed into cuts made on the arm or fist of men in order to make them good boxers. Also, other insect groups like wasps are used for this purpose.

#### Tales and proverbs

There is a tale concerning termites, the hyena and the rabbit and it is called ‘Leuk le Lièvre’ written by Léopold Sédar Senghor, former president of Senegal, and Abdoulaye Sadji [67]. It has to do with the termites having saved ‘Bouki’ the hyena from a trap where he was tied up, by chewing the ropes. Leuk, the hare, knowing that Bouki would be grateful to Mormark the Termite, covered himself with mud just to look like the termite and went to visit Bouki as Mormark. Bouki received him very nicely, gave him food and a room. During the night, there was a rain, the room was unprotected and the mud washed off. Leuk now became the real enemy of Bouki, and was chased away.

Another story is told that Akron, a district of Porto Novo, the capital of Benin, was founded by hunters, who acted on the request of the dwarf ‘Abory Messan Adjadja’ with nine heads, who came out of a termite mound, to construct a temple [68].

A Luo proverb “Biye ojemo ni ng’wen” [69] means “*The fierce white ants cause the death of the kind and harmless ants*” (white ants are termites). “Biye” are the termites that eat and destroy wooden built and grass-thatched Luo houses in East Africa. For that reason, whole termite mounds are dug out and destroyed. However, “ng’wen,” is an ant (*Carebara vidua*) living in nests in close proximity to nests of *Macrotermes* species (*M. natalensis*, *M. michaelseni* and *M. subhyalinus*) [70]. In Africa, between 2 and 10% of *Macrotermes* mounds, contain nests of the ant *C. vidua* also called “Thief ants”. The workers of these ants are minute (less than 2 mm) and carry termite eggs and young through tunnels too small for termites [70]. The young female reproductives of these ants are over 2 cm, and come out of the nests on their nuptial flight after heavy rains. They are popular food throughout Africa and in particular the abdomens, because of the nutritional and medicinal value [71] (Uganda: Ganda, Nyoro); Zimbabwe: Shona, Tonga; Zambia: Nyanja). The abdomens of the large females are eaten raw, roasted or crushed (p.193, [21]), [72, 73]. What the proverbs means is that when a termite nest is destroyed, the nest of the ant is also destroyed and with it a valuable food source. The Luo people in Kenya and Tanzania teach that the elders of families and societies should refrain from indulging in unethical conducts and social misbehaviours during lifetime, as this may affect the prosperity of families and societies.

Another proverb is “Termites cannot eat a stone” (Nigeria: Yoruba): do not do what you cannot handle.

#### Art and literature

It has been suggested that termites, being wood destructors, finish off wooden art in Africa, such as masks, statues and stools. However also archaeology may suffer from the activities of termites, because they change the textural, chemical, mineralogical, and stratigraphic properties of soils. With increased soil porosity bones dissolute more rapidly. Stone artefacts are either dispersed or displaced vertically, complicating the archaeological interpretation [74].

The soil of termites is used to make drawings inside the hut (Burkina Faso: Mossi; Gambia: Jola) or the outer wall (Sudan: Kuku; Uganda: Langi, Luo; Zambia: Lozi, Tonga; Zimbabwe: Ndebele, Shona). The soil used as paint should have a different colour than the wall of the hut.

One book on termites is worth mentioning: the soul of the white ant by Eugene Marais from South Africa (available on line [75], first published as ‘Die Siel van die Mier’ in 1925 in Afrikaans; his work was plagiarised in 1926 by Nobel laureate Maurice Maeterlinck). His theory was that the individual nest of the termites is similar to the organism of an animal: workers and soldiers resembling red and white blood corpuscles, the fungus gardens the digestive organs, the queen functioning as the brain, and the sexual flight being in every aspect analogous to the escape of spermatozoa and ova.

In Africa, termites have been depicted on stamps: termite soldiers (Burkina Faso [76], Republic of the Congo), a queen, a winged reproductive and a worker (Federal Republic of Somalia), a termite mound (the Gabonese Republic, Zimbabwe), an aardvark in front of a termite hill (Ghana), a chimpanzee fishing for termites (Republic of Guinea and Tanzania), and the mushroom *Termitomyces* sp. from Namibia [77].

There are a few poems about termites in Africa which relate to hard working (p. 14, [78]):

- *If anything inspires*
- *Termites inspire even a fool*
- *If people work hard*
- *Termites work even harder*
- *Worse still in the absence of foremen*
- *Tiny in size*
- *They are wiser than Mr gigantic elephant*

## Conclusions

The amazing occurrence of termites in Africa was already expressed in early literature [79]. It is likely that the following characteristics makes them so appealing: the dominance in the landscape, the social organization of the termites, their destructive power, and the provision of food. Termite mounds have even been used biomimetically to design climate control buildings in Zimbabwe, because they have ingenious ventilation systems responsible for steadying the interior temperature [80, 81].

Many articles relate to role of termites in agricultural ecosystems in relation to nutrient cycling, soil turnover, water availability and pest losses [82]. This role of termites is often undervalued [83]. Termites can be manipulated such that crop performance is improved as shown by Mando [84] on Sahelian crusted soils. Termite mounds act as islands of fertility, which are responsible for ecosystem-level spatial heterogeneity in savannas [85]. Mounds of *Ancistrotermes*, *Macrotermes*, *Odontotermes* (family Macrotermitinae), *Cubitermes* (family Termitinae) and *Trinervitermes* (Nasutitermitinae) are significantly enriched in clay (75%), carbon (16%), total nitrogen (42%), calcium (232%), potassium (306%) and magnesium (154%) compared to the surrounding savanna soil. The enrichment of the mounds is even used by mining companies to detect valuable minerals.

Informants hardly mentioned the destructive power of termites or the role as an agricultural pest. The majority of species are economically harmless; of a total of 50 termite genera recorded from southern Africa, only 18 have one or more known pest species [86]. Most considered the beneficial role of termites with regard to soil fertility or their use as food. The religious aspects of mounds (in particular ancestors) was frequently addressed.

In Africa of all insects, termites are probably most popular as food. For example, Niaba et al. (2012) [87] mentions that from 500 people surveyed in Côte d’Ivoire almost all consumed or had consumed termites. In this literature review, 14 species were listed as human food and nine as animal feed in Africa. Termites are harvested from nature and there are many techniques for harvesting the termites and for each species there is a different one [10, 19]. Termites, in particular *Macrotermes* spp. prove to be highly nutritious insects, and a good source of protein, calcium, iron and zinc [88–90]. The relevance is that in Kenya, termites have now been proposed as nutritious food in processed products [88, 91], in particular as complementary food for mothers and children [92]. Most consumers in this country are willing to pay more for termite-based food products provided that the nutritional value is high, food safety is guaranteed and it is officially recommended [93]. The best way to increase the abundance would be to rear the species, but this is extremely difficult and probably not an option. Then the strategy would be to find appropriate techniques to process and preserve the termites to make them continuously available.

What is the relevance of termites as chicken feed in Africa? In Botswana, in commercial poultry production, all the ingredients used in manufacturing feeds are imported causing that feed expenditure account for over 70% of the total production costs [94]. In this country for poultry and in Nigeria for Japanese quail [95]. Also in the DRC, expensive meat meal as protein ingredient in broiler feed could be replaced by meal of collected termites with higher profitability and without compromising weight gain [96]. Nutritionally, termites as a cheap alternative can replace fish meal, but simple rearing, such as indicated in this article, and processing techniques need to be developed.

Termite mounds in many parts of Africa are often associated with ancestors, devils, spirits, witches or ghosts. A question remains whether religion and superstition concerning termites and mounds has a management function, e.g. to protect mounds. According to Taringa [97] the ecological attitude of traditional African religion is more based on fear or respect of ancestral spirits than on respect for nature itself. Not only in Africa but also several parts of India, termite mounds are worshipped [98]. The association of the termite mound, the rain and the rainbow, found in many parts of Africa, seems to be logical as flying termites appear after the first rains.

Termites are intriguing social insects with multiple uses in the physical and spiritual world as reported in this study.

## Abbreviations

CAR: Central African Republic
DRC: Democratic Republic of Congo
RES: Resource persons
Sp(p): Species
